# Delusional Infestation in a Patient with Renal Failure, Metabolic Syndrome, and Chronic Cerebrovascular Disease Treated with Aripiprazole: A Case Report

**DOI:** 10.1155/2011/103652

**Published:** 2011-11-17

**Authors:** Bernardo Carpiniello, Federica Pinna, Raffaella Tuveri

**Affiliations:** Section of Psychiatry & Psychiatric Clinic, Department of Public Health, University of Cagliari, Via Liguria 13, 09127 Cagliari, Italy

## Abstract

Delusional infestation is an aspecific psychiatric condition manifested either as a primary psychotic disorder or a secondary disorder induced by a wide range of very different medical conditions. Both primary and secondary delusional infestations seem to respond to typical and atypical antipsychotics. The latter are considered the first-line treatment although the use of second-generation antipsychotics featuring a higher metabolic, cardiovascular, and renal tolerability is preferable in secondary cases, which often occur in patients with multiple, severe medical conditions. We report a case of a 72-year-old patient affected by delusional infestation associated with severe renal failure, metabolic syndrome, hypertensive cardiopathy, and chronic cerebrovascular disease.

## 1. Background

The term of delusional parasitosis or delusional infestation is used to describe psychotic cases of varying origin. Briefly, the disorder is characterized by the patient's delusional belief that his/her skin and other body areas are infested by small, vivid, or less frequently inanimated pathogens, associated to a series of abnormal body sensations [1, 2]. Two main forms of the disorder have been reported to date, namely, a “primary,” isolated monosymptomatic delusional disorder [3], generally considered a “delusional disorder, somatic type,” according to DSMIVTR [4], and a “secondary” form which may be related to other psychiatric illnesses, toxic or medication-induced psychoses, organic brain disorders, or other general medical conditions. The “secondary” forms are generally, although nonexclusively, included among “psychotic disorder due to general medical condition” according to DSMIVTR [4]. Both “primary” and “secondary” forms seem to respond well to antipsychotic treatment, with either “typical” or “atypical” agents [5–7] although the lack of randomized controlled trials and head-to-head comparisons prevents the drawing of any conclusions as to whether the first-generation drugs are superior in terms of efficacy compared to second-generation antipsychotics [2]. Aripiprazole, an “atypical” antipsychotic, was recently suggested as one of the most promising therapeutical tools in similar cases, mainly due to its pharmacokinetic properties and its fairly good tolerability [8]. Unfortunately, this drug has been used in a limited number of studies to date, and the findings are based upon only a few case reports [9–12]. Here, we report the outcome of a case of delusional infestation in a patient affected by renal failure and chronic cerebrovascular disease treated by means of aripiprazole after an unsuccessful trial with risperidone.

## 2. Case Presentation

A Caucasian male patient, 72 years old, married, and retired, was taken in care in the Community Mental Health Center of Cagliari University (Italy) with a provisional diagnosis of “psychogenic pruritus” upon referral from his family physician. His medical history showed that he had been affected by a chronic nonobstructive pulmonary disease for an unspecified period of time, had manifested insulin-dependent diabetes mellitus type 2 in 1998 and onset of significant renal failure in 2004 (currently on dialysis), and had been diagnosed with hypertension and hypertensive heart disease in 2008. The patient had moreover undergone prostatectomy in 2004 and removal of a non-otherwise-specified “cyst” of the right breast in 2007. At the time of his first appointment, he was taking repaglinide, insulin, calcium acetate, sodium polystyrene, atorvastatin, doxazosin, amlodipine, and clonidine. The family and personal history of psychopathology was totally negative. The symptoms for which he was referred to our outpatient clinic had apparently started following a bout of influenza two years previously (2008). In particular, the patient experienced an intense skin itching associated with the belief that an unspecified insect had laid eggs in various areas of the body, specifically in the skin and in various body cavities (nose, ears, and mouth). Furthermore, he maintained that he could see and feel small animals and pests everywhere although his wife denied ever having seen these animals. The patient was affected by skin lesions due to continuous scratching and manifested an obsessive search for “proof” of infestation, inspecting linens, clothing, and skin folds although he was somewhat upset at not having been ever able to catch the parasites to “show them to the doctors.” The patient was referred to our centre following repeated dermatologic examinations and skin tests, all of which were negative. Significant laboratory findings were mild anaemia (11.4 gr/dL), slight decrease in hematocrit (32.4%), phosphorus (4.8 mg/dL), potassium (5.5 mEq/L), gammaGT (66 U/L), and increases in TRF (193 mg/dL), urea (171 mg/dL), creatinine (6.5 mg/dL) triglycerides (242 mg/dL), ferritin (451.8 ng/mL), and PTH (216 pg/mL) and fasting glucose (212 mg/dL). Levels of folic acid and Vit.B12 were normal, and hepatitis markers were negative. Doppler ultrasound of the supra-aortic vessels showed an atherosclerotic plaque located on the proximal left common carotid causing a local stenosis of 35% and other nonstenosing plaques on the middle part of the left common carotid and carotid bifurcation bilaterally. The ECG was normal except for a slight left axis deviation (QRS axis <20). Echocardiogram revealed a slight thickening of the left ventricle, while aortic arch and ascending aorta were at the upper limit of normality, and a minimal tricuspid insufficiency and a mild aortic valve sclerosis were detected with a normal systolic function (EF 60%). Chest X-ray showed a small fibrotic nodule in the left lateral-basal area, and a flattening of the diaphragm. CT scan of the brain revealed the presence of residual signs of a left, nucleocapsular ischemic lesion, while MRI showed different areas of altered signal, hyperintense in FLAIR, and T2 and DP, in the periventricular white matter, in frontoparietal subcortical left and right areas and semioval centers due to chronic vascular hypoperfusion, in addition to expansion of the ventricular system and of cortical and cisternal spaces due to cerebral atrophy. Psychiatric evaluation confirmed the presence of a pervasive delusion of infestation associated with somatic and visual hallucinations. Nonsignificant cognitive impairment on Mini Mental State Examination (MMSE) was evidentiated (total score = 27). From the end of July 2010, the patient was treated with oral risperidone, 1 mg initially; after approximately a month, the dose was increased to 2 mg due to scarce efficacy. Approximately one month later, a slight improvement was observed (delusions were less pervasive and disturbing, and itching was reduced). However, after two months of treatment, risperidone was gradually withdrawn due to an increasing sedation and to the absence of further clinical improvement. As a replacement oral aripiprazole was gradually introduced, 10 mg daily initially, being increased after 2 weeks to 15 mg daily. After four weeks, the clinical picture was considerably improved, with disappearance of delusions, hallucinations, and itching, without recovery of insight. Probably due to this reason, for some months the patient made repeated requests to discontinue therapy, deeming it useless. He accepted, however, to continue, maintaining the same dosage as maintenance therapy. The only side effect attributable to the introduction of aripiprazole appeared after the first two weeks of treatment, in terms of a midterminal insomnia, which required additional treatment with quetiapine 12.5 mg for approximately a month; after this period, the drug was suspended, but sleep remained fairly good. Over the following months, no recurrence of psychotic symptoms was observed, but lack of retrospective insight remained. Accordingly, and given the fact that therapy was well tolerated (no significant side effects, modification of laboratory test, or ECG were detected), it was decided to continue maintenance treatment with aripiprazole 15 mg daily. After 10 months of treatment, the patient is in a stable condition.

## 3. Discussion

The case described could be considered a secondary form of delusion of infestation, in the light of the presence of several major diseases which have been reported in literature as associated with delusional parasitosis although in practice it can sometimes be difficult to determine whether a case is a primary monosymptomatic delusional disorder or secondary to an organic pathology. Indeed, a seminal review undertaken by by Freundenmann and Lepping [2] reports 6 cases associated with hypertension, 23 cases associated with other forms of cerebrovascular disease (mainly arteriosclerosis), 7 cases associated with diabetes mellitus, and 3 cases associated with renal failure; moreover, a recent case in a patient affected by renal failure and cerebral atrophy was described by Tzeng and Chiang [12]. Naturally, in a case such as that here described, characterized by multiple medical diseases, it is impossible to determine exactly which disease may have triggered the onset of delusional disorder. Notwithstanding the limits of our knowledge on the effectiveness of antipsychotic drugs, based almost solely on case series and case reports, both primary and secondary forms of the disorder seemed to respond to the administration of either typical or atypical antipsychotics [5–7], emphasizing no well-defined superiority of one over the other. In this case, as the patient was aged and featured multiple problems, the administration of pimozide and haloperidol had been ruled out, particularly in view of their acknowledged issues relating to cardiovascular tolerability. Thus, risperidone was selected, largely due to its low-risk profile in patients with heart disease, metabolic syndrome, or renal failure and to reports maintaining the higher efficacy of risperidone in secondary forms [2]. Unfortunately, the lack of a significant clinical response within two months and the problems of tolerance (excess of sedation) induced us to replace risperidone treatment. Aripiprazole was subsequently prescribed on the basis of its good tolerability profile with regard particularly to the cardiovascular, metabolic, and renal function. An increased adherence of the patient to treatment was moreover hoped for in view of the good tolerability of the drug. The switch was effective, and a remission of psychotic symptoms was achieved in approximately two months. This timespan was in agreement with the average of approximately six weeks reported in literature as being necessary to achieve similar results [2] although Tzeng and Chiang [12] reported an extremely rapid clinical response (approximately two weeks) in a very similar case. At clinical regimen, the dose administered was 15 md a day, in line with other reports [9, 11, 13, 14] prescribing doses ranging between 10 and 30 mg, but in contrast with the low dose (5 mg) used in the above-cited case by Tzeng and Chiang [12] and with the case described by Freudenrich et al. [15]. On continuation of treatment, minimal transient side effects were reported (insomnia), thus confirming the efficacy and tolerability profile reported previously, even in patients with particularly compromised general conditions such as the case described here [12, 15]. However, it should be underlined that clinical remission in the patient studied cannot be considered complete, as he continues to lack any true retrospective insight. This finding is supported by reports present in literature which have demonstrated that even patients treated successfully with pimozide, considered the gold standard treatment for this condition for a considerable period, failed to recover their insight [14]. 
